# L2-L1 Translation Priming Effects in a Lexical Decision Task: Evidence From Low Proficient Korean-English Bilinguals

**DOI:** 10.3389/fpsyg.2018.00267

**Published:** 2018-03-02

**Authors:** Yoonhyoung Lee, Euna Jang, Wonil Choi

**Affiliations:** ^1^Department of Psychology, Yeungnam University, Kyoungsan, South Korea; ^2^Division of Liberal Arts and Sciences, Gwangju Institute of Science and Technology, Gwangju, South Korea

**Keywords:** L2-L1 translation priming, Korean-English unbalanced bilinguals, masked priming, bilingual word recognition, lexical decision task

## Abstract

One of the key issues in bilingual lexical representation is whether L1 processing is facilitated by L2 words. In this study, we conducted two experiments using the masked priming paradigm to examine how L2-L1 translation priming effects emerge when unbalanced, low proficiency, Korean-English bilinguals performed a lexical decision task. In Experiment 1, we used a 150 ms SOA (50 ms prime duration followed by a blank interval of 100 ms) and found a significant L2-L1 translation priming effect. In contrast, in Experiment 2, we used a 60 ms SOA (50 ms prime duration followed by a blank interval of 10 ms) and found a null effect of L2-L1 translation priming. This finding is the first demonstration of a significant L2-L1 translation priming effect with unbalanced Korean-English bilinguals. Implications of this work are discussed with regard to bilingual word recognition models.

## Introduction

A crucial question on understanding bilinguals' lexico-semantic organization is how the native (L1) and the second language (L2) interact with each other. In particular, researchers have focused on the issue of how L2 (or L1) words are mapped onto meaning in bilingual word recognition (De Groot and Kroll, 2014). One way to examine this issue is to use the translation priming paradigm in which a prime word presented briefly in one language is followed by a target word in another language. Analyzing the pattern of the priming effect allows for understanding the nature of the lexical organization of bilinguals.

A substantial body of research has shown that priming effects are obtained in the direction of L1 to L2 (forward priming), in which an L2 target word is responded to faster when preceded by its translation (e.g., 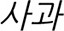
—*apple*) than by an unrelated L1 word [e.g., 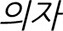 *(chair in English)*—*apple*; e.g., De Groot and Nas, 1991; Williams, 1994; Gollan et al., 1997; Jiang, 1999; Jiang and Forster, 2001; Kim and Davis, 2003; Basnight-Brown and Altarriba, 2007; Voga and Grainger, 2007; Duyck and Warlop, 2009; Dimitropoulou et al., 2011a,b; Lupker et al., 2015]. This effect has been robust and found not only between similar script pairs (e.g., Dutch-English, Spanish-English, and Spanish-Catalan), but also between different script pairs (e.g., Chinese-English, Japanese-English, and Korean-English), suggesting that the L1-L2 translation priming effect should occur irrespective of orthographic similarities between two languages (for a meta-analytic review, see Wen and van Heuven, 2017). Moreover, the forward priming effect is observed in conditions using the masked priming technique in which the prime word is preceded by a forward mask (e.g., ######) and prime duration is very short (Wen and van Heuven, 2017). This result suggests that the forward priming effect is a product of automatic language processing, and is not a strategic effect.

In contrast to these findings, a backward translation priming effect (L2 prime-L1 target) tends to be very weak or even absent especially for the different-script non-cognate translation equivalents. The main focus of the current study is on non-cognate translation pairs, which refer to words that share the same meanings but do not share orthographical or phonological similarity (e.g., Gollan et al., 1997; Jiang, 1999; Jiang and Forster, 2001; Finkbeiner et al., 2004; Duyck and Warlop, 2009; Schoonbaert et al., 2009; Davis et al., 2010; Dimitropoulou et al., 2011a; cf., Dimitropoulou et al., 2011b; Witzel and Forster, 2012; Nakayama et al., 2013; Wang, 2013; Chen et al., 2014; Wang and Forster, 2015). The asymmetry in the priming effect between forward and backward directions provides useful insight for the development of bilingual word recognition models. It is also noteworthy that this asymmetry occurs robustly in unbalanced bilinguals (Gollan et al., 1997; Jiang, 1999; Finkbeiner et al., 2004; Nakayama et al., 2013). For balanced bilinguals who are fluent in both languages, the asymmetry has been found to decrease or even to disappear completely (Perea et al., 2008; Duñabeitia et al., 2010a,b). In a recent review paper, Wen and van Heuven (2017) conducted a meta-analysis of 64 masked priming lexical decision experiments to evaluate effect sizes of the forward and backward translation priming effects in bilinguals. They found that effect size was significantly larger for forward than for backward priming.

This asymmetry of the L1-L2 (or L2-L1) translation priming effect can be explained by examining models of bilingual word recognition. One explanation comes from the episodic L2 hypothesis, proposed by Jiang and Forster (2001, see also Witzel and Forster, 2012). According to this hypothesis, L1 words are represented in lexical memory whereas L2 words are stored in episodic memory (except in cases where bilinguals acquired meaning with both languages simultaneously in their childhood), which suggests that L2 representations are distinct from L1 representations. Therefore, L1 prime words can activate lexical memory, which facilitates processing of L2 translation equivalents; in contrast, L2 primes do not facilitate processing of the lexically-represented L1 translation equivalents. The episodic L2 hypothesis is based on the results from Jiang and Forster (2001). They used an episodic recognition task, in which participants first learned a list of L1 (Chinese) words (a study phase), followed by a recognition memory test (i.e., old/new decision) where an L2 (English) masked prime word was briefly presented before an L1 target word. Results showed that reaction time for the recognition memory test was faster in the L2 translation prime than in the L2 unrelated prime condition only when L1 target words were learned at the study phase. Note that these results were obtained with Chinese-English bilinguals who were native speakers of Mandarin and started learning English from middle school in Mainland China. This result was replicated by Witzel and Forster (2012)^1^.

The L1-L2 translation priming asymmetry can also be explained by the sense model, developed by Finkbeiner et al. (2004). The sense model suggests that L2 words are incapable of activating sufficient semantic sense to facilitate the processing of L1 translation equivalents. However, L1 primes can activate an ample amount of semantic sense that can be utilized to facilitate L2 target processing. In a lexical decision task, Finkbeiner et al. showed a significant priming effect in the L1-L2 direction but no effect in the L2-L1 direction with Japanese-English proficient bilinguals who were tested in the United States (see also Xia and Andrews, 2015). Note that the episodic L2 hypothesis and the sense model both assume that the asymmetry occurs due to imbalances between the two languages. In the case of proficient, balanced, or early bilinguals, L2-L1 translation priming could be produced either because L2 words are stored in lexical memory, rather than episodic memory, or because L2 words can activate sufficient semantic sense to facilitate L1 processing (Basnight-Brown and Altarriba, 2007; Wang, 2013; Nakayama et al., 2016).

Another model that might explain the asymmetric translation priming effect is the Distributed Representation Model (DRM, de Groot, 1992a,b; see also Schoonbaert et al., 2009 for a refined version of the model). The refined DRM assumes that cross-linguistic priming effects should be influenced by the magnitude of semantic overlap between the L1 and the L2 word. Schoonbaert et al. (2009) reported that intermediate Dutch-English bilinguals showed effects of both translation priming and semantic priming in the L2-L1 direction as well as in the L1-L2 direction at stimulus onset asynchrony (SOA) of both 100 and 250 ms, using a 50 ms prime presentation rate. Although the effect size was larger in the L1-L2 direction, the L2-L1 priming effect was still significant. These results can be accounted for by the refined DRM, which posits that the lexico-semantic system of the two languages is quantitatively different, rather than qualitatively different.

In addition, the Bilingual Interactive Activation plus (BIA+) model (Dijkstra and Van Heuven, 2002) can explain the translation priming asymmetry. The BIA+ model proposes two systems: a word identification system and a task/decision system^2^. The BIA+ model assumes that orthographic, phonological, and semantic representations of the two languages are integrated in the word identification system, which forms an interactive network among the representations. Another assumption of the BIA+ model is that access to the bilingual mental lexicon occurs in a language-independent way. In other words, a word presented in either language can activate sublexical and lexical representations in both languages. Although the two languages' orthographic, phonological, and semantic features can be activated in parallel, initial levels of activation differ based on the resting-level activation of lexical items. For example, higher frequency words have higher resting-level activation values compared to lower frequency words. In addition, highly proficient bilinguals have higher resting-level activation in their L2 words compared to less proficient bilinguals. Accordingly, activation of lexical representations of L2 words can be delayed relative to that of L1 words for unbalanced bilinguals, which implies that unbalanced bilinguals need more time to activate proper lexical representations of L2 words than those of L1 words based on the resting-level activation of lexical items as well as on the degree of L2 proficiency.

The BIA+ model not only explains the asymmetrical patterns of L1-L2 translation priming, but also addresses the possibility that the asymmetrical priming effect could be modulated by the aforementioned variables (resting-level activation of words and L2 proficiency). In the masked priming paradigm, L2 prime words are perhaps presented too briefly (around 50 ms) for unbalanced bilinguals to sufficiently activate lexico-semantic representations of the corresponding target in L1. With respect to L2 proficiency, the BIA+ model proposes that resting activation levels increase as L2 proficiency of bilinguals increases, which might lead to a significant L2-L1 translation priming effect in the masked priming paradigm. For example, Nakayama et al. (2016) recently showed that highly proficient Japanese-English bilinguals obtained a robust L2-L1 translation priming effect whereas less proficient bilinguals failed to obtain the same effect when 50 ms of SOA (and prime duration) was used in their masked priming experiments. This suggests that the L2-L1 translation priming effect may emerge if the resting activation levels of L2 words are increased.

Another possible way to modulate the L2-L1 translation priming effect would be to manipulate the duration between prime and target. According to the BIA+ model, the null effect of L2-L1 translation priming is due to lower levels of resting activation. By increasing the duration to process an L2 prime word, the L2 words may be more strongly activated, leading to a significant L2-L1 translation priming effect. Indeed, although most studies using a masked priming paradigm with a prime duration of around 50 ms have failed to observe L2-L1 translation priming effects even with highly-proficient bilinguals (see Duñabeitia et al., 2010b; Nakayama et al., 2016, for a summary), Basnight-Brown and Altarriba (2007) reported a significant L2-L1 translation priming effect with a masked priming paradigm in which a 100 ms prime duration was implemented. Basnight-Brown and Altarriba demonstrated an important role of prime duration in modulating the L2-L1 translation priming effect with highly-proficient English-Spanish bilinguals who learned both languages relatively early in their life (before 6 years old on average). Thus, we do not know if the results were due to the increased prime duration or to the level of L2 proficiency of the participants (note that in this study the prime was clearly visible at a presentation rate of 100 ms). An important question is therefore whether the L2-L1 translation priming effect can be modulated by prime and/or SOA duration even in unbalanced bilinguals.

In addition to Basnight-Brown and Altarriba (2007), recent studies have shown that the L2-L1 translation priming effect emerges in a lexical decision task with unbalanced bilinguals with lower levels of L2 proficiency (Duyck and Warlop, 2009; Schoonbaert et al., 2009; Dimitropoulou et al., 2011b). For example, Duyck and Warlop (2009) observed a significant L2-L1 priming effect using 112 ms SOA (56 ms prime duration followed by 56 ms backward mask) with unbalanced Dutch-French bilinguals. As described earlier, Schoonbaert et al. (2009) also found a meaningful L2-L1 priming effect using the 100 and 250 ms SOA (50 ms prime duration in both SOAs) in a lexical decision task with unbalanced Dutch-English bilinguals.

Although these two studies showed L2-L1 translation priming effects in low-proficient L2 learners who spoke languages with the same script, there are scarce studies that have examined this issue when the languages do not share the script. Indeed, the studies conducted thus far with low-proficient L2 learners who spoke languages with a different script have used a 50 ms SOA and have obtained inconsistent results (e.g., null effect in Nakayama et al., 2016 with Japanese-English bilinguals and a translation L2-L1 priming effect in Dimitropoulou et al., 2011b with Greek-English bilinguals). Therefore, it is crucial to further examine the role of SOA duration in L2-L1 translation priming effect with low-proficient L2 learners who speak languages with a different script, as we have done in the present research. Note there are studies showing that the differences in writing systems could influence word recognition. For example, Wang et al. (2003) showed that Korean-English beginning bilinguals performed better than Chinese-English beginning bilinguals in a phoneme deletion task in which subjects were asked to delete a specific phoneme in a real English word to make another English word. Wang et al. argued that Korean learners were better at this task than Chinese learners because the writing systems of Korean and English are more similar than the writing systems of Chinese and English. These results imply that dis/similarity of writing systems between languages could affect bilingual word recognition.

The current study used a masked lexical decision paradigm with systematic variations in SOA while maintaining the prime duration constant (50 ms). Specifically, we used two SOAs (60 and 150 ms). The long SOA condition (150 ms) was chosen to ensure participants had enough time to process the prime stimulus without adopting any conscious strategies. Although Basnight-Brown and Altarriba (2007) found significant translation priming effects with an SOA of 100 ms, the participants recruited in that study were highly proficient bilinguals. In contrast, the participants in the current study were unbalanced low proficiency bilinguals. Thus, we increased the SOA to 150 ms to allow these less proficient participants enough time to process the word. The rationale was that this longer SOA would increase the probability of obtaining the L2-L1 priming effect while also minimizing the possibility that participants might draw on conscious strategies. It is worth noting here that there are some studies showing null effects of translation priming in the long SOAs (longer than 150 ms) with different scripts (e.g., Chinese-English in Jiang and Forster, 2001). However, the null effects could be due to low power such that the number of items per cell might not be enough (Wen and van Heuven, 2017). The current study will use greater number of items per cell to increase power.

From a theoretical perspective, the main goal of the current study was to examine how L1/L2 lexical representations are stored and processed during word recognition. As described earlier, the BIA+ model provides a reasonable explanation for why proficient bilinguals showed a significant L2-L1 translation priming effect using the masked-priming technique. According to the BIA+ model, bilinguals' two languages are integrated in a single system so that the L2-L1 translation priming effect can occur in cases where L2 words are effectively activated (e.g., Nakayama et al., 2016). If this is the case, it would be expected that a significant L2-L1 translation priming effect should be observed when a long SOA (around 150 ms) is implemented even in unbalanced bilinguals because the long SOA can provide enough time to strongly activate L2 primes. However, other models such as the episodic L2 hypothesis or the sense model assume that unbalanced bilinguals' mental lexicons of two languages are qualitatively different. Accordingly, they predict that there should be no L2-L1 translation priming effect when the long SOA condition is used.

## Experiment 1: L2-L1 translational priming with a 150 ms SOA

In the current study, two experiments were conducted to examine the role of SOA in the L2-L1 masked translation priming effect while maintaining prime duration (50 ms) constant. In experiment 1, 150 ms SOA was used to test whether the L2-L1 translation priming effect would emerge with unbalanced low proficient Korean-English bilinguals.

### Method

#### Participants

Thirty native speakers of Korean^3^ with English as a second language participated in the current experiment. All subjects gave informed consent, following the ethics protocol approved by the Institutional Review Board of the Yeungnam University. They were unbalanced, low proficiency, Korean-English bilinguals, who started to learn English later in their life (after 10 years old). English proficiency was assessed with TOEIC (Test of English for International Communication) scores. In addition, self-report measures on L2 experience were collected from participants. As an objective measure of L2 proficiency, we used total TOEIC scores of reading and listening. The TOEIC has been the most widely used test in Korea to differentiate English proficiency of non-native speakers of English. As measures of L2 experience, two self-report questionnaires were used. One questionnaire was used to report participants' overall previous experience with English. For this questionnaire, we used a 5-point Likert scale (1 = no experience; 5 = extreme experience). The other questionnaire assessed how frequently participants use English in everyday life. We asked participants to circle “never,” “sometimes,” or “always”. The summarized results for the L2 proficiency of the participants are shown in Table 1. Results from these questionnaires reveal that participants rarely used English in their everyday lives, even though they had a certain extent of experience in English. This suggests that participants' experience with English was likely acquired through formal education. Participants received course credit for their participation.

**Table 1:** Mean English proficiency scores from experiment 1.

**TOEIC score**	**Self-rated experience in English (5 point scale)**				**Daily usage (3 point scale)**	
	**Reading**	**Listening**	**Speaking**	**Writing**	**Use of Korean**	**Use of English**
655.47 (160.05)	3.00 (1.06)	2.69 (0.98)	2.18 (0.98)	2.20 (3.30)	3.00 (0.15)	1.62 (0.53)

*Standard deviations are provided in parentheses*.

#### Materials

Sixty Korean words were used as targets. Target words were between one and three syllables in length (*M* = 1.98, *SD* = 0.5). Mean word frequency ranged from 8 to 71,306 (*M* = 4,620, *SD* = 10,636), calculated based on the Sejong corpus (Kang and Kim, 2009). One hundred twenty English words were used as primes. Half were the English translation equivalents of the Korean target words, while the other half were unrelated words that served as primes for the control condition. The English translation primes had a mean log frequency of 3.29 (Brysbaert and New, 2009) and were on average 5.02 letters long (range: 4–6). The unrelated control primes were matched to the translation primes in mean log frequency (3.28) and letter length (*M* = 5.00, range: 4–6), *t*s < 1. In addition, concreteness ratings of English prime words for the translation vs. unrelated condition were examined using norms provided by Brysbaert et al. (2014). The mean concreteness ratings for the translation condition (3.78) and the unrelated condition (3.86) were not statistically different, *t*_(59)_ = −0.43, *p* > 0.05. All prime-target pairs are listed in the Appendix.

We manipulated the prime words to vary the prime-target relationship. Two counterbalanced lists were created so that a target word was preceded by a translation equivalent prime word in one list, while the same target was preceded by an unrelated prime word in the other list. Each participant was assigned to only one list (60 prime-target pairs) and saw target words only once. To help mask the purpose of the experiment, 42 prime (L2) and target (L1) pairs that were not semantically or phonologically related to experimental primes and targets were used as filler trials. In addition, because 102 word targets were presented in each list, 102 Korean pronounceable non-word targets were also included in each list. All Korean non-word targets were preceded by English word primes.

#### Procedure

Before starting the experiment, participants gave informed consent. After participants were given the instructions and indicated that they fully understood what to do, they were presented with a training block of 10 trials. After the training block, participants were presented with an experimental block, which took about 10 min. Each trial had the following sequence: a forward mask (#######) was presented in the center of the screen for 500 ms and was replaced by a prime stimulus. The prime stimulus was presented for 50 ms and was immediately followed by a blank screen for 100 ms. A target word then appeared and remained on the screen until participants made a lexical decision. Participants were instructed to press the “/” key on a keyboard if the letter string was a word and the “z” key if the letter string was a non-word. When participants made their decision, the next trial was automatically initiated. Stimuli presentation was controlled using E-prime 2.0 (Psychology Software Tools Inc., 2012).

### Results and discussion

Mean response times (RTs) and error rates are shown in Table 2. Incorrect responses were excluded from the analysis of response times (1.1%). In addition, RTs beyond two standard deviations were excluded from the analyses (4.3%). In total, 5.4% of data were not included in the analyses. An ANOVA, with the prime type as an explanatory variable (translation equivalent vs. unrelated), was conducted to test whether the RTs of lexical decisions for target words varied as a function of the prime type. Analyses were performed with participants (*F*_1_) and items (*F*_2_) as random variables. The exact same selection criteria were also used for the subsequent experiment in this study.

**Table 2:** Mean response times and error rates from experiment 1.

	**RTs (ms)**	**Error rates (%)**
Translation	529 (37)	1.3 (0.02)
Unrelated	539 (37)	1.1 (0.02)

*Standard deviations are provided in parentheses*.

There was a significant main effect of the prime type, showing that RTs in the translation prime condition were faster than those in the control prime condition, *F*_1(1, 29)_ = 11.89, *p* < 0.005, *MSE* = 133, *d* = 0.62; *F*_2(1, 59)_ = 4.47, *p* < 0.05, *MSE* = 623; *d* = 0.27^4^. This result demonstrates that participants made a lexical decision faster for the target words when they were preceded by their translation equivalent compared to when they were not. The ANOVA on the error rates failed to show the priming effect, *F*_s_ < 1, ns.

The reaction time results showed a significant L2-L1 translation priming effect such that the lexical decision latency for L1 target words was shorter when preceded by the L2 translation equivalent relative to when preceded by an unrelated L2 prime word. Given that most previous studies have found no translation priming effect with low/intermediate bilinguals when using a 50 ms SOA (e.g., Exp. 3 and 4 in Gollan et al., 1997; Exp. 3 in Jiang and Forster, 2001; Exp. 3 in Nakayama et al., 2016; Exp. 1 in Wang, 2013), this result indicates that increasing SOA helps unbalanced bilinguals activate lexico-semantic features of L2 words, resulting in the facilitation of processing L1 translation equivalent words. In addition, given that many studies have failed to observe an L2-L1 translation priming effect with unbalanced different-script bilinguals (for summarized results of recent studies, see Table 1 in Nakayama et al., 2016; cf., Duyck and Warlop, 2009; Dimitropoulou et al., 2011b), we argue that the significant L2-L1 translation priming effect obtained in the current experiment results from the use of a longer SOA. In experiment 2, we use a short SOA (60 ms) to test this idea.

## Experiment 2: L2-L1 translational priming with a 60 ms SOA

In Experiment 1, we observed a significant L2-L1 translation priming effect with a 150 ms SOA. In Experiment 2, we tested whether the priming effect observed in Experiment 1 was driven by the use of a long SOA (150 ms). To this end, in Experiment 2 we used a 60 ms SOA. If the priming effect reported in Experiment 1 is due to the use of a long SOA, we would expect a null effect of L2-L1 translation priming in Experiment 2. Alternatively, if the priming effect occurred due to another factor, like participants' L2 proficiency (although we classified the participants recruited in Experiment 1 as low proficiency bilinguals, their L2 proficiency might be higher than we thought), then we would expect a priming effect to emerge even with a short SOA.

### Method

#### Participants

Forty-three native speakers of Korean with English as a second language participated in the current experiment. All subjects gave informed consent, following the ethics protocol approved by the Institutional Review Board of the Yeungnam University. The participants were unbalanced bilinguals. Their previous experience with English, as assessed through questionnaires, is shown in Table 3. Note that the English proficiency of the participants in Experiment 2 was very similar to that in Experiment 1. Participants received course credit for their participation.

**Table 3:** Mean English proficiency scores from experiment 2.

**TOEIC score**	**Self-rated experience in English (5 point scale)**				**Daily usage (3 point scale)**	
	**Reading**	**Listening**	**Speaking**	**Writing**	**Use of Korean**	**Use of English**
665.71 (129.83)	3.04 (0.78)	2.69 (0.86)	2.23 (0.93)	2.38 (0.85)	3.00 (0.21)	1.81 (0.45)

*Standard deviations are provided in parentheses*.

#### Materials

The same stimuli used in Experiment 1 were also used in this experiment.

#### Procedure

In Experiment 2, we used a 10 ms blank duration rather than 100 ms. Thus, a forward mask (#######) was presented in the center of the screen for 500 ms, and was replaced by a prime stimulus. The prime stimulus was presented for 50 ms, immediately followed by a 10 ms blank screen. Then a target word appeared after the blank screen. Otherwise, the procedure for Experiment 2 was identical to the procedure for Experiment 1.

### Results and discussion

Incorrect responses were excluded from the analysis of response times (0.7%). In addition, RTs beyond two standard deviations were excluded from the analyses (3.7%). In total, 4.4% of the data were not included in the analyses. Mean response times (RTs) are shown in Table 4. Neither RTs^5^ nor error rates between the translation and the unrelated condition were statistically significant, *F*s < 1, ns.

**Table 4:** Mean response times and error rates from experiment 2.

	**RTs (ms)**	**Error rates (%)**
Translation	621 (112)	0.8 (0.02)
Unrelated	625 (120)	0.8 (0.01)

*Standard deviations are provided in parentheses*.

The current experiment used a 60 ms SOA which has been used in most previous L2-L1 translation priming studies using the masked priming paradigm. As observed in the previous studies, we obtained a null effect of translation priming in the L2-L1 direction. Given this result, the significant translation priming effect observed in Experiment 1 appears to result from a longer SOA.

## General discussion

A significant L2-L1 translation priming effect in a masked priming lexical decision task has rarely been observed in the extant literature. The present study examined whether the L2-L1 translation priming effect would emerge in low proficiency unbalanced Korean-English bilinguals by manipulating the duration of the SOA (60 vs. 150 ms) while holding the prime duration constant (50 ms).

The results were straightforward: we found a significant L2-L1 translation priming effect when a 150 ms SOA was used. However, no significant effect was observed when a 60 ms SOA was used. Taken together, these results indicate that the null effect observed in previous studies, and in Experiment 2 of the current study, is likely due to the use of an insufficient SOA (i.e., below 100 ms).

The results reported here are the first to show different-script L2-L1 translation priming with low proficiency unbalanced Korean-English bilinguals. Kim and Davis (2003) recruited unbalanced Korean-English bilinguals and reported an L1-L2 translation priming effect, but they did not report the results of the L2-L1 direction. Although research on bilingual lexico-semantic representation with different-script bilinguals has been conducted with many different language combinations such as Chinese-English or Japanese-English, studies with Korean-English bilinguals are still very scarce.

The current results fit well within the BIA+ framework, suggesting that the L2-L1 translation priming effect can be influenced by the SOA and the L2 proficiency. As mentioned earlier, according to the BIA+ model, the emergence of an L2-L1 translation priming effect is dependent upon the strength of the resting level activation for words in the word identification system of the model. Thus, the priming effect can be obtained in cases where the resting level activation for L2 words is as strong as that for L1 words, such as when the L2-L1 translation priming effect emerges in studies with *balanced* bilinguals (e.g., Basnight-Brown and Altarriba, 2007; Duñabeitia et al., 2010b; Dimitropoulou et al., 2011b; Wang, 2013; Sabourin et al., 2014). In addition, proficient *unbalanced* bilinguals have also shown an L2-L1 translation priming effect (Nakayama et al., 2016). Within the framework of the BIA+ model, these results indicate that L2 proficiency can modulate the resting level for L2 words, affecting the occurrence of an L2-L1 translation priming effect. In other words, because bilinguals with low levels of L2 proficiency have low resting activation levels for L2 words, they may not demonstrate a translation priming effect from a very brief exposure of L2 prime words. Indeed, our results suggest that SOA is a critical factor modulating the L2-L1 translation priming effect. A priming effect was observed when a 150 ms SOA was used, whereas a null effect was found when a 60 ms SOA was used. This pattern suggests that increasing the SOA also raises the activation strength for L2 words in the word identification system of the BIA+ model, which leads to a rapid response to an L1 translation equivalent relative to a control word. Importantly, the L2-L1 translation priming effect was observed even in unbalanced, low proficiency bilinguals. A goal for future research should be to examine the relationship between SOA and L2-L1 translation priming in low proficient bilinguals or L2 learners at a more fine-grained level.

The current results can be also explained by the refined DRM (Schoonbaert et al., 2009). According to this model, the degree to which priming effects emerge depends upon the magnitude of conceptual nodes shared by prime and target words. Therefore, the L2-L1 translation priming effect observed in Experiment 1 (150 ms SOA) is consistent with this model. The proportion of semantic nodes connected to an L2 translation target is larger than the proportion of nodes connected to an L2 unrelated target, which leads to a larger priming effect in the translation condition than in the unrelated condition. The null effect of translation priming reported in Experiment 2 (60 ms SOA) might then be because the 60 ms duration is not long enough to activate semantic nodes to make the translation priming effect emerge. Schoonbaert et al. also reported a numerically larger effect size for translation priming at a 250 ms SOA vs. a 100 ms SOA, suggesting that the duration between prime and target is a critical factor influencing patterns of cross-linguistic priming effects.

The results reported here are not compatible with either the Sense model (Finkbeiner et al., 2004) or the episodic L2 hypothesis (Jiang and Forster, 2001; Witzel and Forster, 2012). As mentioned in the Introduction, these two models assume that L2 words are processed qualitatively differently from L1 words. According to the Sense model, L2 words only activate a small portion of senses at a lexico-semantic level. Thus, increasing the SOA is not enough to activate a full range of senses of the words. Likewise, according to the episodic L2 hypothesis, L2 words are processed in episodic memory whereas L1 words are processed in the lexical memory system^6^. Thus, manipulation of the SOA would not be expected to change the general pattern of the L2-L1 priming effect, at least for unbalanced, low proficiency bilinguals. However, the present study did show such a priming effect at an SOA of 150 ms. Again, this suggests that the results reported here are not consistent with either of these two models.

One thing to note is that several previous studies using an SOA longer than the one used in Experiment 1 of the current study have still reported a null L2-L1 translation priming effect. For example, Xia and Andrews (2015) found a null L2-L1 translation priming effect in unbalanced Chinese-English bilinguals with moderate to high proficiency in L2 using a 200 ms SOA with 50 ms prime duration (see also Jiang, 1999; Jiang and Forster, 2001; Witzel and Forster, 2012; Chen et al., 2014). We propose several possible reasons for this inconsistency.

One reason could be the characteristics of the writing system of the native language. The null effect observed in the previous studies was obtained from unbalanced Chinese-English bilinguals, whereas the significant L2-L1 translation priming effect reported in the current study was observed from unbalanced Korean-English bilinguals. Substantial evidence shows that the processes of L2 word recognition are influenced by L1 linguistic knowledge, which is referred to as L1 transfer (van Heuven et al., 1998; Jared and Kroll, 2001; Wang et al., 2003; Choi et al., 2015). In other words, linguistic knowledge about the L1 writing system can affect the processes of L2 word recognition especially when the two languages' writing systems have similar properties (Wang et al., 2003). Accordingly, the inconsistent results between the previous studies using unbalanced Chinese-English bilinguals and the current study using unbalanced Korean-English bilinguals might be dependent upon how L1 knowledge (e.g., Chinese or Korean) affects L2 (e.g., English) word recognition. This possibility should be systematically investigated in future research.

Another possible reason might be the number of items used for the experiments. Previous studies reporting a null effect for L2-L1 priming using English-Chinese translation pairs have frequently used fewer than 16 items per cell in a factorial design (e.g., Gollan et al., 1997; Jiang, 1999; Witzel and Forster, 2012; Chen et al., 2014; Xia and Andrews, 2015; cf. Wang, 2013), whereas the current study used 30 items per cell. In a recent meta-analysis, Wen and van Heuven (2017) statistically evaluated the effect sizes of L1-L2 and L2-L1 translation priming effects. They showed that the effect size of the L1-L2 direction was larger than that of the L2-L1 direction. In particular, the meta-analysis revealed that the effect size of the L2-L1 translation priming effect was modulated by the number of items per cell. Brysbaert and Stevens (2018) have also recommended that at least 1,600 observations (e.g., 40 participants and 40 items per condition) are needed to achieve adequate statistical power for these experiments.

Two methodological limitations of the current study are worth noting. First, we did not measure prime visibility in either experiment. If a prime word were visible to participants, they might notice the relationship between primes and targets, which might then allow participants to recruit strategic processes to complete the lexical decision task. However, given the short prime duration of 50 ms and the participants' low proficiency in L2, this possibility seems unlikely. Indeed, some previous studies have reported no effect of proportion relatedness when similar SOAs were used (e.g., Perea and Rosa, 2002). Second, in our experimental procedures, a blank screen was presented between the prime and target, which could make the prime stimulus more salient, and therefore might increase the possibility that participants could process the prime consciously. Note that a recent ERP study also inserted a blank period between prime and target in order to maximize the size of the priming effects (Jiang, 1999; Gutierrez-Sigut et al., 2017). An interesting goal for future research is to carefully examine how differences in priming techniques might modulate the magnitude of priming effects.

In summary, we demonstrated a significant L2-L1 non-cognate translation priming effect in which unbalanced, low proficiency Korean-English bilinguals performed a masked priming lexical decision task with a 150 ms SOA. This finding is easily explained within the BIA+ model or DRM. Although there have been many studies that have examined cross-script bilinguals' lexico-semantic organization using the masked priming paradigm with translation equivalent pairs, the current study is the first demonstration of L2-L1 translation priming using unbalanced low proficiency Korean-English bilinguals.

## Author contributions

YL: Designing experiments, interpreting results, and writing a manuscript. EJ: Selecting materials, conducting experiments, and writing a manuscript. WC: Conceptualization, designing experiments, interpreting results, and writing a manuscript.

### Conflict of interest statement

The authors declare that the research was conducted in the absence of any commercial or financial relationships that could be construed as a potential conflict of interest.

## Figures and Tables

**Table A1:**
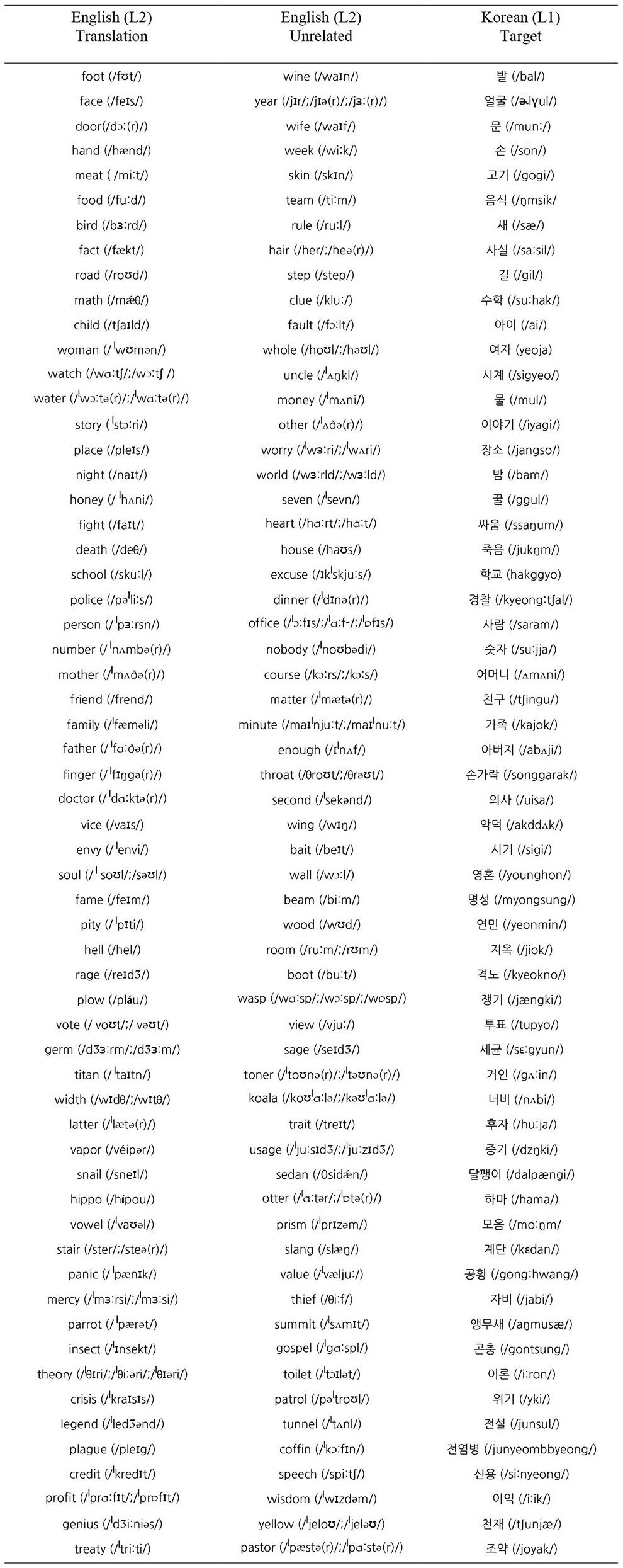
Non-cognate translation prime words, unrelated prime words, and target words used in Experiments 1 and 2.

## Footnotes

^1^Witzel and Forster (2012) and Jiang and Forster (2001) both used highly proficient bilinguals as participants.

^2^In this paper, we describe the word identification system in detail. Detailed description of the task/decision system is beyond the scope of the current paper. For a more comprehensive description for the BIA+ model, see Dijkstra and Van Heuven (2002).

^3^Because the participants in both experiments were native speakers of Korean, we did not evaluate their L1 (Korean) proficiency. They were born in South Korea, and their parents were also native speakers of Korean, meaning that their home language is Korean. Therefore, the participants recruited in both experiments were highly proficient and homogeneous with respect to L1 proficiency.

^4^Following a reviewer's suggestion, we conducted *post-hoc* power analyses of the RT analyses using G^*^Power 3. 1.9.2 (Faul et al., 2009). The statistical power (1-β) of the *F*_1_ analysis was ~1 and that of the *F*_2_ analysis was 0.84. Although the *F*_2_ analysis showed less power than the *F*_1_ analysis (Brysbaert and Stevens, 2018), both analyses had a reasonable level of power.

^5^Following a reviewer's suggestion, we conducted Bayes Factor analyses using JASP (JASP Team, 2018) to better understand this null effect. The Bayes factor (BF_01_) for the F1 analysis was 5.18, meaning that there is 5.18:1 evidence in favor of the null hypothesis. The Bayes factor (BF_01_) for the F2 analysis was 6.89. These results indicate that the null effect of Experiment 2 is not likely to be due to low power (Type II error).

^6^Nakayama et al. (2016) proposed that the episodic L2 hypothesis could explain a significant L2-L1 translation priming effect in unbalanced bilinguals as their proficiency increases. This proposal assumes that the processes of L2 word recognition occur in lexical memory, rather than in episodic memory.
